# Association of unmet basic resource needs with frailty and quality of life among older adults with cancer—Results from the CARE registry

**DOI:** 10.1002/cam4.6038

**Published:** 2023-05-28

**Authors:** Grant R. Williams, Mackenzie Fowler, Smith Giri, Chen Dai, Christian Harmon, Mustafa Al‐Obaidi, Coryn Stephenson, Kira Bona, Wendy Landier, Smita Bhatia, Julie Wolfson

**Affiliations:** ^1^ Institute for Cancer Outcomes & Survivorship University of Alabama Birmingham Alabama USA; ^2^ O'Neal Comprehensive Cancer Center University of Alabama Birmingham Alabama USA; ^3^ University of Missouri Columbia Missouri USA; ^4^ Division of Population Sciences Dana‐Farber Cancer Institute Boston Massachusetts USA

**Keywords:** aging, basic resource needs, cancer, concrete resource needs, frailty, geriatric oncology

## Abstract

**Background:**

Basic resource needs related to transportation, housing, food, and medications are important social determinants of health and modifiable indicators of poverty, but their role in modifying the risk of frailty and health‐related quality of life (HRQoL) remains unknown. The goal of our study was to examine the prevalence of unmet basic needs and their association with frailty and HRQoL in a cohort of older adults with cancer.

**Methods:**

The CARE registry prospectively enrolls older adults (≥60 years) with cancer. Assessments of transportation, housing, and material hardship were added to the CARE tool in 8/2020. The 44‐item CARE Frailty Index was used to define frailty, and subdomains of physical and mental HRQoL were assessed using the PROMIS® 10‐global. Multivariable analysis examined the association between unmet needs with frailty and HRQoL subdomains, adjusting for covariates.

**Results:**

The cohort included 494 participants. Median age of 69 years, 63.6% were male and 20.2% were Non‐Hispanic (NH) Black. Unmet basic needs were reported in 17.8% (transportation 11.5%, housing 2.8%, and material hardship 7.5%). Those with unmet needs were more often NH Black (33.0% vs. 17.8%, *p* = 0.006) and less educated (<high school: 19.5% vs. 9.7%, *p* = 0.023). Compared to those without unmet needs, unmet needs were associated with higher odds of frailty (adjusted odds ratio [aOR] 3.3, 95% CI 1.8–5.9), low physical (aOR = 2.1, 95% CI 1.2–3.8) and low mental (aOR = 2.5, 95% CI 1.4–4.4) HRQoL.

**Conclusions:**

Unmet basic needs represent a novel exposure that is independently associated with frailty and low HRQoL and warrants the development of targeted interventions.

## 1 INTRODUCTION

Despite advances in cancer prevention, detection, and management over the past several decades, disparities in cancer outcomes persist across the cancer continuum.^1^ Nonbiological correlates of health outcomes, commonly known as social determinants of heath, are associated with cancer outcomes,^2^ and increasingly recognized as important targets for reducing health disparities.^3^ Unmet basic resource needs related to transportation, housing, food, and medications are important and modifiable indicators of poverty.^4^ Prior work among adults with chronic cardiometabolic diseases demonstrates that targeting basic unmet needs can lead to tangible improvements in health outcomes.^5^ Although poverty is associated with inferior health outcomes among patients with cancer, most studies have relied on area‐level measures of poverty (e.g., at the county level) to approximate individual‐level poverty.^6^, ^7^, ^8^ Examination of “current poverty” (≥20% of population living in poverty) and/or “persistent poverty” (≥20% of population living in poverty for three consecutive decades) has yielded consistent and strong associations with increased mortality and inferior cancer outcomes. Nevertheless, both the concrete driver(s) of these poverty‐related findings and the role of individual‐level factors in these associations remain unknown.

There is a dearth of information regarding the prevalence of unmet basic resource needs among older adults with cancer, which is crucial as older adults represent the majority of new cancer diagnoses and cancer deaths.^9^ The management of cancer in older adults is often complicated by the coexistence of age‐related impairments and comorbid conditions. Frailty is a recognized state of increased vulnerability and is prevalent in older adults with cancer.^10^, ^11^ Frailty is associated with increased chemotherapy toxicities, hospitalizations, long‐term care placement and reduced health‐related quality of life (HRQoL), and inferior survival.^12^, ^13^, ^14^, ^15^ Socioeconomic status are known to contribute to frailty in the general population, but the relationship of frailty with poverty among older adults with cancer remains uncertain.^16^, ^17^, ^18^ The role of unmet basic resource needs, self‐identified unmet resource needs related to transportation, housing, food, utilities, and medications/medical care, in modifying the risk of frailty among older adults with cancer remains unknown.

We address these knowledge gaps by examining the prevalence of unmet basic resource needs and its association with frailty and HRQoL in a cohort of older adults with cancer.

## 2 METHODS

### 2.1 Study population

The Cancer and Aging Resilience Evaluation (CARE) study at the University of Alabama at Birmingham (UAB) is a registry of older adults (≥60 years) seen at UAB Hospital and Clinics for their cancer care; enrollment began in 2017, and a brief social determinants of health section was added to the core tool in August 2020.^19^ We included adults 60 years or older, given the uncertainty of the appropriate chronologic age cutoff for “older” adults and among CARE participants, there is a similar prevalence of age‐related impairments and frailty among those 60–65 years and those 65–75 years and > 75 years.^20^ For this study, we included the subset of participants recruited between August 2020 and April 2022 that completed the social determinants of health section. This study was approved by the Institutional Review Board at University of Alabama at Birmingham (IRB‐300000092) and performed in accordance with the ethical standards of the 1964 Declaration of Helsinki and its later amendments.

### 2.2 Study measures

#### 2.2.1 Basic resource needs

Social determinants of health measures embedded within the CARE tool assessed basic needs as outlined here: (1) Health‐related transportation insecurity was measured using two previously published items:^21^ *How much trouble is it for you to get transportation to your doctor?* [dichotomized as *some trouble/a lot of trouble* versus *a little trouble/no trouble*]; *Have you ever missed a doctor's appointment because of transportation issues*? [*yes/no*]. Patients were classified as having unmet transportation needs if they identified trouble in either of these questions. (2) Housing insecurity was assessed using two items adapted from the Protocol for Responding to and Assessing Patients Assets, Risks and Experiences (PRAPARE) instrument developed by the National Association of Community Health Centers.^22^ The first inquired about the participant's current housing situation [*I have housing; I do not have housing* (*staying with others, in a hotel, in a shelter, living outside on the street, on a beach, in a car, or in a park*)] and the second asked whether participants were worried about losing their housing [*yes/no*].^23^ Patients were classified as having unmet housing needs if they responded *I do not have housing* or had concerns about losing their housing. (3) Material hardship (security of food, utilities, and medications/medical care) was assessed using a single item adapted from the PRAPARE instrument: *In the past year, have you or any family members you live with been unable to get any of the following when it was really needed?* [a list of yes/no items was provided: *food, utilities, medicine or any healthcare (medical, dental, mental health, vision), phone, clothing, child care, or other*].^23^ Patients were classified as having unmet material needs if they answered yes to any of the items. Patients were classified as having overall unmet basic resource needs if they had any unmet needs in one or more of the categories above (transportation, housing or material hardship).

#### 2.2.2 Frailty

Using the principles of deficit accumulation and following the procedures outlined by Searle et al.,^24^ we constructed the CARE Frailty Index.^25^ Based on 44 identified health deficits from the CARE geriatric assessment tool, the CARE Frailty Index was calculated as the proportion of deficits for each patient (range 0–1). Participants were required to have nonmissing data for at least 30 items in order to compute a valid frailty score and were categorized as robust (0–0.2), prefrail (0.2–0.35), and frail (>0.35), as previously described.^24^ Our team has previously shown that the CARE frailty index predicts functional decline, severe chemotherapy toxicities, and survival among older adults;^25^ similarly constructed frailty indices have shown comparable results.^12^, ^13^, ^14^, ^15^, ^26^ See Table S1 for a full list of the CARE frailty index items.

#### 2.2.3 Health‐related quality of life

The CARE tool assesses HRQoL using the National Institutes of Health Patient‐Reported Outcomes Measurement Information System® (PROMIS®) Global Health 10‐item short‐form. The PROMIS Global Health 10‐item scale includes separate scoring for physical and mental health subscales.^27^, ^28^ PROMIS measures have been tested in large samples of adults in the United States and item responses are converted to t‐scores with a standardized mean score of 50 and a standard deviation of 10.^30^ The minimal clinically relevant difference for PROMIS ranges from 2 to 6 points, and a score of ≤40 (1 standard deviation) is considered impaired for the subscales.^29^ Low physical and mental subdomains of HRQoL were defined as a t‐score of ≤40 (1 standard deviation).

#### 2.2.4 Covariates

Patients self‐reported information regarding race, ethnicity, education, marital status, and employment. Urban–rural status was obtained via patient‐reported ZIP code merged with Rural–Urban Commuting Area (RUCA) code data. Categorization B from the University of Washington School of Medicine was used to define urban, micropolitan, and rural status.^30^, ^31^ Urban and micropolitan were combined into one urban group due to similarity in outcomes between the two as discussed in a prior study.^32^ Information regarding cancer stage, cancer type, and date of diagnosis were abstracted from the electronic medical record.

### 2.3 Statistical analyses

Distribution‐appropriate bivariate statistical tests, namely chi‐squared test/Fisher's exact test for categorical variables, were used to compare patient characteristics and frailty categories between those with and without unmet basic resource needs. Logistic regression models were used to evaluate the association between unmet basic resource needs with frailty and physical and mental domains of HRQoL. An additional logistic regression model was used to assess predictors of unmet basic resource needs. Multivariable models were adjusted for potential confounders including age, sex, race/ethnicity, education, marital status, employment status, urban–rural status, cancer type, and cancer stage. All hypothesis testing was two‐sided and the level of significance was set at 0.05. All statistical analyses were conducted using SAS statistical software version 9.4 (SAS Institute Inc.).

## 3 RESULTS

### 3.1 Patients

Between August 2020 and April 2022, a total of 494 older adults completed the CARE tool and the social determinants of health survey (see Figure S1 for consort diagram). Most participants were age 60–69 years (56.2%), male (63.6%), and non‐Hispanic White (75.1%) (Table 1). Most participants had a high school education or some college (52.9%), were married (57.6%), and were retired (62.1%). Most participants resided in urban (90.7%) versus rural areas (9.3%). Finally, participants had either colorectal cancer (33.1%), pancreatic (18.0%), hepatobiliary (12.0%), or other cancers (36.9%). The majority had advanced stage disease (stage III: 30.6%; stage IV: 44.5%). Overall, 29.0% of the cohort was frail (see Table S1 for prevalence of individual frailty item impairments); impaired physical HRQoL was observed in 36.2% and impaired mental HRQoL in 39.1%. Median time from cancer diagnosis to CARE tool completion was 35 days.

**TABLE 1:** Participant characteristics.

Variable	Total	Unmet need, 88 (17.8%)	No unmet needs, 406 (82.2%)	*p*‐value
Age group				0.630
60–64	147 (29.9)	30 (34.1)	117 (29.0)	
65–69	129 (26.3)	26 (29.6)	103 (25.6)	
70–74	102 (20.8)	14 (15.9)	88 (21.8)	
75–79	57 (11.6)	9 (10.2)	48 (11.9)	
80+	56 (11.4)	9 (10.2)	47 (11.7)	
Sex, male	314 (63.6)	52 (59.1)	262 (64.5)	0.336
Race/Ethnicity				**0.021**
Non‐Hispanic White	364 (75.1)	56 (64.4)	308 (77.4)	
Non‐Hispanic Black	98 (20.2)	27 (31.0)	71 (17.8)	
Other	23 (4.7)	4 (4.6)	19 (4.8)	
Education				**0.023**
<High school	56 (11.4)	17 (19.5)	39 (9.7)	
High school	148 (30.2)	31 (35.6)	117 (29.0)	
Some college	111 (22.7)	18 (20.7)	93 (23.1)	
Associate's/Bachelor's degree	101 (20.6)	13 (14.9)	88 (21.8)	
Advanced degree	74 (15.1)	8 (9.2)	66 (16.4)	
Employment status				**<0.001**
Retired	303 (62.1)	50 (56.8)	253 (63.3)	
Disabled	67 (13.7)	23 (26.1)	44 (11.0)	
Part‐time (<32 h/week)	12 (2.5)	3 (3.4)	9 (2.3)	
Full‐time (≥32 h/week)	62 (12.7)	3 (3.4)	59 (14.8)	
Other	44 (9.0)	9 (10.2)	35 (8.8)	
Marital status				**<0.001**
Single	46 (9.4)	18 (20.9)	28 (6.9)	
Widowed/Divorced/Separated	162 (33.1)	37 (43.0)	125 (30.9)	
Married	282 (57.6)	31 (36.1)	251 (62.1)	
Urban–rural residence				0.465
Urban	448 (90.7)	78 (88.6)	370 (91.1)	
Rural	46 (9.3)	10 (11.4)	36 (8.9)	
Cancer type				**<0.001**
Colorectal	162 (33.1)	25 (28.7)	137 (34.0)	
Pancreatic	88 (18.0)	16 (18.4)	72 (17.9)	
Hepatobiliary	59 (12.0)	22 (25.3)	37 (9.2)	
Other	181 (36.9)	24 (27.6)	157 (39.0)	
Cancer stage				0.675
0–II	117 (24.9)	23 (26.4)	94 (24.5)	
III	144 (30.6)	29 (33.3)	115 (30.0)	
IV	209 (44.5)	35 (40.2)	174 (45.4)	
Frailty	142 (29.0)	47 (54.0)	95 (23.6)	**<0.001**
Impaired physical HRQoL	167 (36.2)	48 (57.1)	119 (31.5)	**<0.001**
Impaired mental HRQoL	189 (39.1)	53 (61.6)	136 (34.2)	**<0.001**

*Note*: Bold values indicate significance at < 0.05.

### 3.2 Basic unmet needs

**FIGURE 1:**
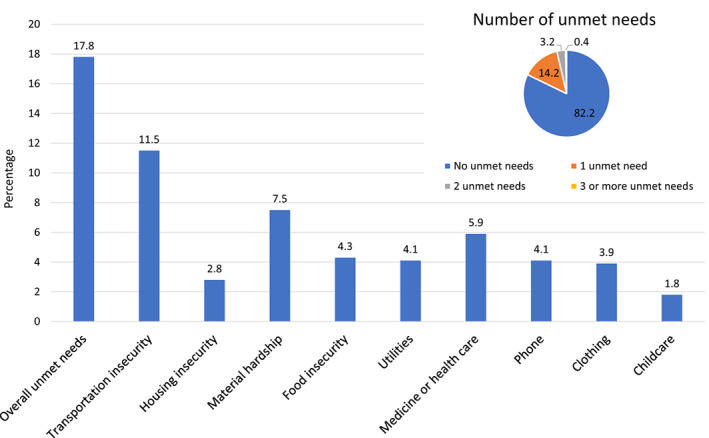
Prevalence of basic unmet needs.

Overall, 17.8% of patients were classified as having a basic unmet need; 14.2% had an unmet need in one category, 3.2% in two categories and 0.4% in three categories (see Figure 1). Transportation insecurity was the most prevalent unmet need at 11.5%, followed by material hardship at 7.5% (subcategories including food: 4.3%, utilities: 4.1%, medicine/health care: 5.9%, phone: 4.1%, clothing: 3.9%, childcare: 1.8%) and housing insecurity at 2.8%.

### 3.3 Patients with and without basic unmet needs

When compared with those without any basic unmet need, there was an over‐representation of non‐Hispanic Black (31% vs. 17.8%, *p* = 0.021), lower education (high school: 35.6% vs. 29.0%, *p* = 0.023), those who were disabled (26.1% vs. 11.0%, *p* < 0.001), and those who were unmarried (single: 20.9% vs. 6.9%; widowed/divorced/separated: 43.0% vs. 30.9%, *p* < 0.001) among patients with any basic unmet need (Table 1). In addition, there was also a higher proportion of participants with pancreatic or hepatobiliary cancers (pancreatic: 18.4% vs. 17.9%; hepatobiliary: 25.3% vs. 9.2%, *p* < 0.001) among those with basic unmet needs. However, there was no difference by cancer stage (*p* = 0.675). Finally, those with any unmet need were more likely to be frail (54.0% vs. 23.6%, *p* < 0.001) and have impaired physical (57.1% vs. 31.5%, *p* < 0.001) and mental (61.6% vs. 34.2%, *p* < 0.001) HRQoL. Predictors of having any basic unmet need included non‐Hispanic Black relative to non‐Hispanic White race (odds ratio [OR]: 2.1, 95% CI: 1.2, 3.5), being single (OR: 5.2, 95% CI: 2.6, 10.5) or widowed/divorced relative to married (OR: 2.4, 95% CI: 1.4, 4.0), and having less than a high school education relative to an advanced degree (OR: 3.60, 95% CI: 1.42, 9.10), and having hepatobiliary relative to colorectal cancer (OR: 3.3, 95% CI: 1.7, 6.4) (Table 2).

**TABLE 2:** Unadjusted odds ratios of demographics and clinical variables to basic unmet needs.

Demographics	Unadjusted odds (95% CI)
Age group	
60–64	REF
65–69	0.98 (0.55, 1.77)
70–74	0.62 (0.31, 1.24)
75–79	0.73 (0.32, 1.66)
80+	0.75 (0.33, 1.69)
Sex	
Female	REF
Male	0.79 (0.50, 1.27)
Race	
Non‐Hispanic White	REF
Non‐Hispanic Black	**2.09 (1.24, 3.54)**
Other	1.16 (0.38, 3.53)
Educational level	
Less than high school	**3.60 (1.42, 9.10)**
High school graduate	2.19 (0.95, 5.03)
Some college	1.60 (0.66, 3.89)
Associate/Bachelors	1.22 (0.48, 3.11)
Advanced Degree	REF
Marital status	
Married	REF
Single	**5.21 (2.59, 10.48)**
Widowed/Divorced	**2.40 (1.42, 4.04)**
Urban–Rural status	
Urban	REF
Rural	1.32 (0.63, 2.77)
Clinical	
Cancer type	
Colorectal	REF
Pancreatic	1.22 (0.61, 2.43)
Hepatobiliary	**3.26 (1.65, 6.42)**
Other	0.84 (0.46, 1.53)
Cancer stage	
I/II	REF
III	1.03 (0.56, 1.90)
IV	0.82 (0.46, 1.47)

*Note*: Bold values indicate significance at < 0.05.

Abbreviation: CI, confidence interval.

### 3.4 Multivariable analysis

**FIGURE 2:**
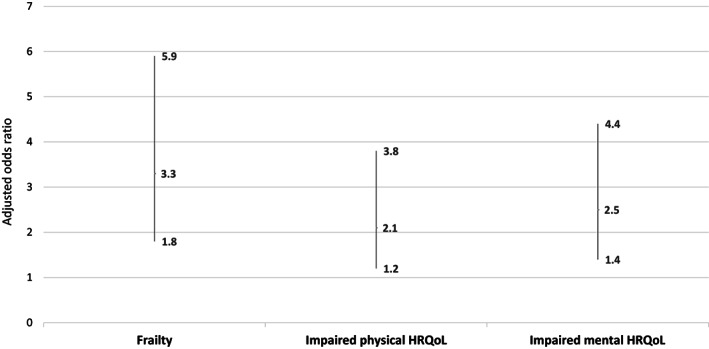
Multivariable logistic regression of the association between basic unmet needs with frailty and reduced physical and mental health‐related quality of life.

Having any basic unmet need was associated with 3.3‐fold higher adjusted odds of frailty compared to not having a basic unmet need (adjusted OR [aOR] 3.3, 95% CI: 1.8, 5.9) after adjustment for age, sex, race/ethnicity, education, employment status, marital status, urban–rural status, cancer type, and cancer stage (Figure 2 and Table S2). Additionally, having any basic unmet need was associated with 2.1‐fold higher odds of physical (aOR 2.1, 95% CI: 1.2, 3.8) and 2.5‐fold higher odds of mental (aOR 2.5, 95% CI: 1.4, 4.4) impaired HRQoL after adjusting for these variables (see Figure 2 and Table S3).

## 4 DISCUSSION

Here, we use a unique prospectively assembled registry of older adults with cancer to examine the association between basic unmet resource needs and frailty and HRQoL, and reveal that unmet needs were associated with a 3.3‐fold higher adjusted odds of frailty and more than a twofold higher adjusted odds of impaired physical and mental HRQoL. Our findings are consistent with the notion that social determinants of health are associated with frailty and HRQoL among older adults with cancer. The most prevalent unmet need was transportation insecurity, with many reporting missing appointments due to transportation difficulties. In addition, several participants reported material insecurities including food insecurity or difficulty obtaining medicine or health care.

The prevalence of unmet basic needs in our study is lower than expected compared with prior studies. In one of the largest intervention studies to date by Berkowitz et al, 34.6% of adults within a primary care network in the Boston metropolitan area screened positive for an unmet need.^5^ In families of pediatric cancer survivors, household material hardship, defined as insecurity of food, housing, or energy, had a similar prevalence of 32%.^33^ More specifically, the prevalence of food insecurity in our study was lower than anticipated based on the literature. Prior estimates range widely from 8% to 55% of patients with cancer experiencing food insecurity depending on the setting, with those having the highest prevalence from low‐income and underserved urban communities.^34^, ^35^ Previous studies have found similar associations with food insecurity, including non‐Hispanic Black race and lower education.^36^ While the prevalence of transportation barriers was the highest among the examined basic needs in our study, the prevalence of transportation issues was also lower than the literature suggests.^37^ Similarly, the prevalence of housing insecurity varies by population, but is often higher than the 3% we found.^38^ The lower prevalence of unmet needs in our study of older adults may be explained in part by a trend toward increased basic unmet needs among younger patients in these prior studies.^34^, ^38^, ^39^ In addition, most studies of individual social determinants (transportation, housing, and food insecurity) are focused on the social determinants as their primary outcome and thus can utilize longer, multi‐item questionnaires to determine insecurity; on the contrary, our study integrated an abbreviated survey into a more global registry survey, which is likely less sensitive.^35^ Furthermore, although our study population is from the US Deep South, an area notable for health disparities, this sample is from a single large academic medical center and not representative of the entire region nor directly comparable to many of the prior studies from urban areas of the northeastern United States.^4^, ^5^, ^33^ Of note, UAB Hospital and Clinics provide care for all patients irrespective of insurance and/or immigration status, and prior research from the CARE Registry demonstrated a mix of 51.5% Medicare, 2.7% Medicaid, 3.6% uninsured/self‐pay, and 42.3% private insurance.^40^

Although socioeconomic conditions are known to contribute to the incidence of frailty, this study is among the first to examine the association of basic unmet needs, recognized as concrete, remediable indicators of poverty, with frailty among older adults with cancer.^16^, ^17^, ^18^ While we cannot demonstrate causality within the context of our cross‐sectional study, several plausible mechanisms exist to suggest that unmet needs may lead to increased frailty. First, unmet basic needs are likely associated with less access to medical care and reduced preventative care, thus resulting over time in increased rates of frailty.^4^, ^41^ Second, insecurity of basic needs may contribute to higher rates of mental distress and stress, and in turn, this chronic stress may result in increased frailty.^42^, ^43^ Lastly, the mental distress and stress related to unmet needs may increase risk behaviors related to frailty (e.g., smoking and alcohol consumption).^44^, ^45^

Interventions related to social determinants of health have been effective in improving health outcomes across health conditions.^46^ More specifically, interventions to address unmet basic resource needs such as food, housing, or medications have improved clinical outcomes.^5^ For example, addressing unmet basic needs among 1700 study participants resulted in improvements in blood pressure and cholesterol levels.^5^ Similar multicomponent interventions, that include social determinants of health, have demonstrated effectiveness in improving diabetes outcomes and HRQoL.^47^ The existing strategies vary substantially in terms of involved workforce (professional vs. lay), setting (community vs. clinic or hospital based), and length of interaction (episodic vs. longitudinal).^4^, ^5^ While further work is necessary to determine the most applicable strategy to address the unmet needs of older adults doing so may prove useful in improving cancer outcomes.

One of the populations identified with the most unmet needs were Black participants. Racial disparities in cancer outcomes are well‐recognized, yet the underlying causes remain an area of ongoing focus. The illustrated differences in unmet basic needs by race, may in part, explain some outcome disparities. Black participants within the CARE registry report a higher prevalence of financial distress, which is intrinsically related to the basic unmet needs.^40^ However, while racial disparities in frailty among older adults with cancer have been demonstrated, unmet needs nevertheless remained significantly associated with increased frailty even after controlling for race.^48^ The growing evidence suggest that some combination of unmet needs, financial strain, and low socioeconomics likely contribute to racial disparities in health outcomes; thus, identifying the appropriate target amenable to intervention among these domains is a promising avenue toward reducing racial disparities and promoting health equity.

Our study is not without limitations. As our analyses are cross‐sectional in nature, no causality or directionality can be drawn between associations. Our study population was from a single center in the Southeastern United States and may not be representative of other older adult populations. Our study relies on patient‐reported measures of health, including for the report of unmet needs and the geriatric assessment information, and lacks additional objective assessment to collaborate these reports. However, the use of patient‐reported outcomes measures has expanded dramatically over the last decade and has been shown to be frequently more accurate than provider or caregiver reports.^49^, ^50^ And although the CARE Registry has a high enrollment proportion (approximately 80%),^19^ there remains some potential for selection bias.

Assessing social determinants of health within oncology care identifies critical and potentially remediable basic unmet needs that may be important drivers of poor outcomes among older adults with cancer. Investigating interventions to address basic unmet needs will be critical to improving HRQoL and reducing the adverse outcomes associated with frailty in vulnerable older adults with cancer. Although high‐quality interventional studies on these unmet needs are lacking, assessing and addressing these basic unmet needs in oncology practice in the short term makes intuitive sense, and may improve the outcomes of our most vulnerable cancer populations.

## AUTHOR CONTRIBUTIONS

**Grant R Williams:** Conceptualization (equal); data curation (equal); formal analysis (equal); funding acquisition (equal); methodology (equal); writing – original draft (equal); writing – review and editing (equal). **Mackenzie E Fowler:** Conceptualization (equal); data curation (equal); formal analysis (equal); methodology (equal); writing – original draft (equal); writing – review and editing (equal). **Smith Giri:** Conceptualization (equal); methodology (equal); writing – review and editing (equal). **Chen Dai:** Data curation (equal); formal analysis (equal); writing – review and editing (equal). **Christian Harmon:** Data curation (equal); writing – review and editing (equal). **Mustafa AL‐Obaidi:** Data curation (equal); writing – review and editing (equal). **Coryn Stephenson:** Investigation (supporting); writing – review and editing (equal). **Kira Bona:** Conceptualization (equal); writing – review and editing (equal). **Wendy Landier:** Conceptualization (equal); writing – review and editing (equal). **Smita Bhatia:** Conceptualization (equal); methodology (equal); supervision (equal); writing – review and editing (equal). **Julie A. Wolfson:** Conceptualization (equal); methodology (equal); writing – review and editing (equal).

## FUNDING INFORMATION

Supported in part by the National Cancer Institute of the National Institutes of Health (K08CA234225) and the Doris Duke Charitable Foundation CARES program at UAB. The content is solely the responsibility of the authors and does not necessarily represent the official views of the National Institutes of Health. Previously presented at the American Society of Clinical Oncology (ASCO) 2022 annual meeting.

## CONFLICT OF INTEREST STATEMENT

The authors declare no competing financial interests.

## ETHICS APPROVAL

Approved by the IRB at UAB (IRB‐300000092) and patients all consented.

## Supporting information


Figure S1.

Table S1.
Table S2.
Table S3.

## Data Availability

Study data are available upon request to the corresponding author.
