# Supplementary figures and images for: Association of unmet basic resource needs with frailty and quality of life among older adults with cancer—Results from the CARE registry
Source: Cancer Med. 2023 May 28;12(12):13846–55. doi: 10.1002/cam4.6038 (PMC10315805; doi:10.1002/cam4.6038)

## Slide 1
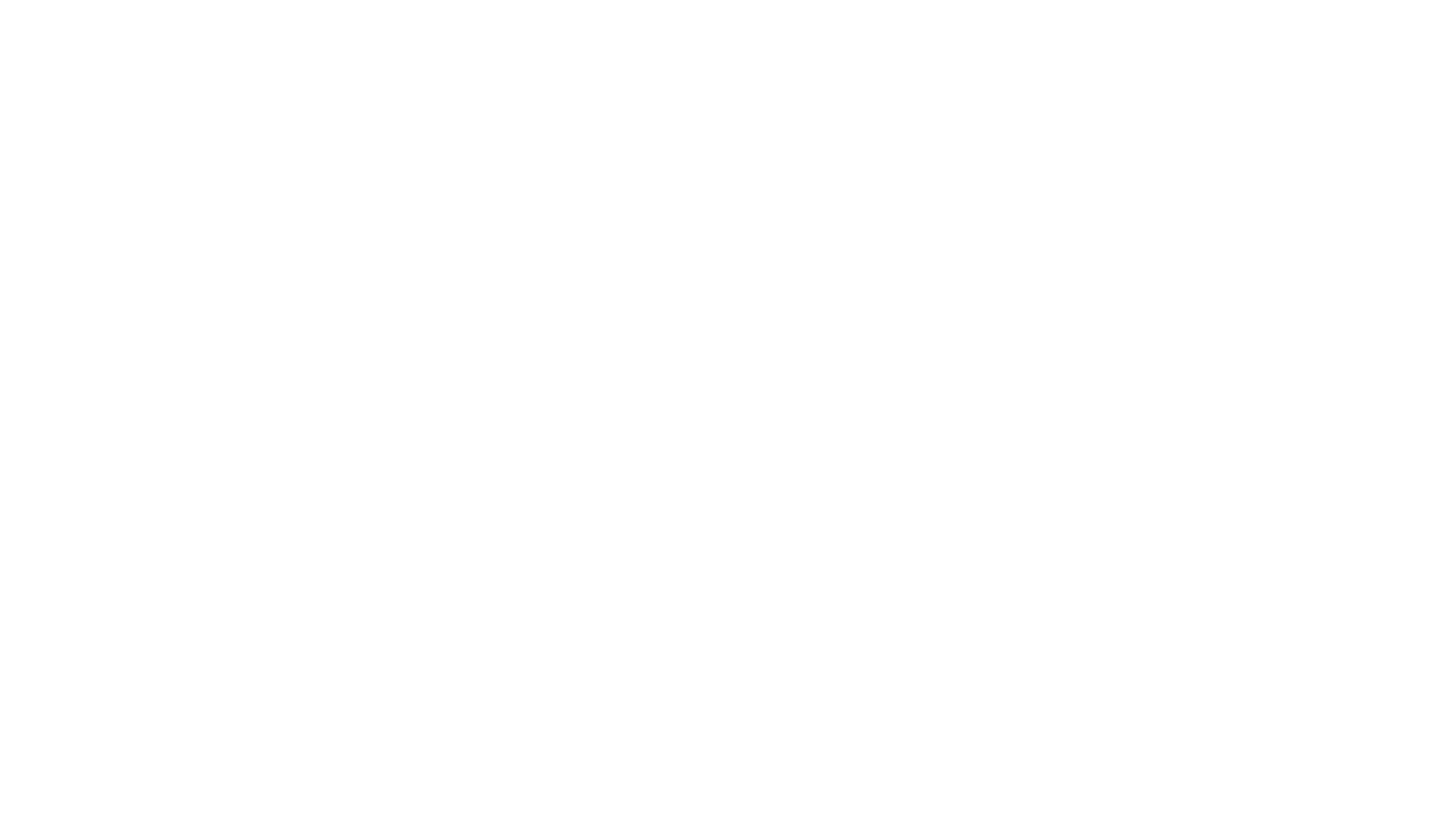

## Slide 2
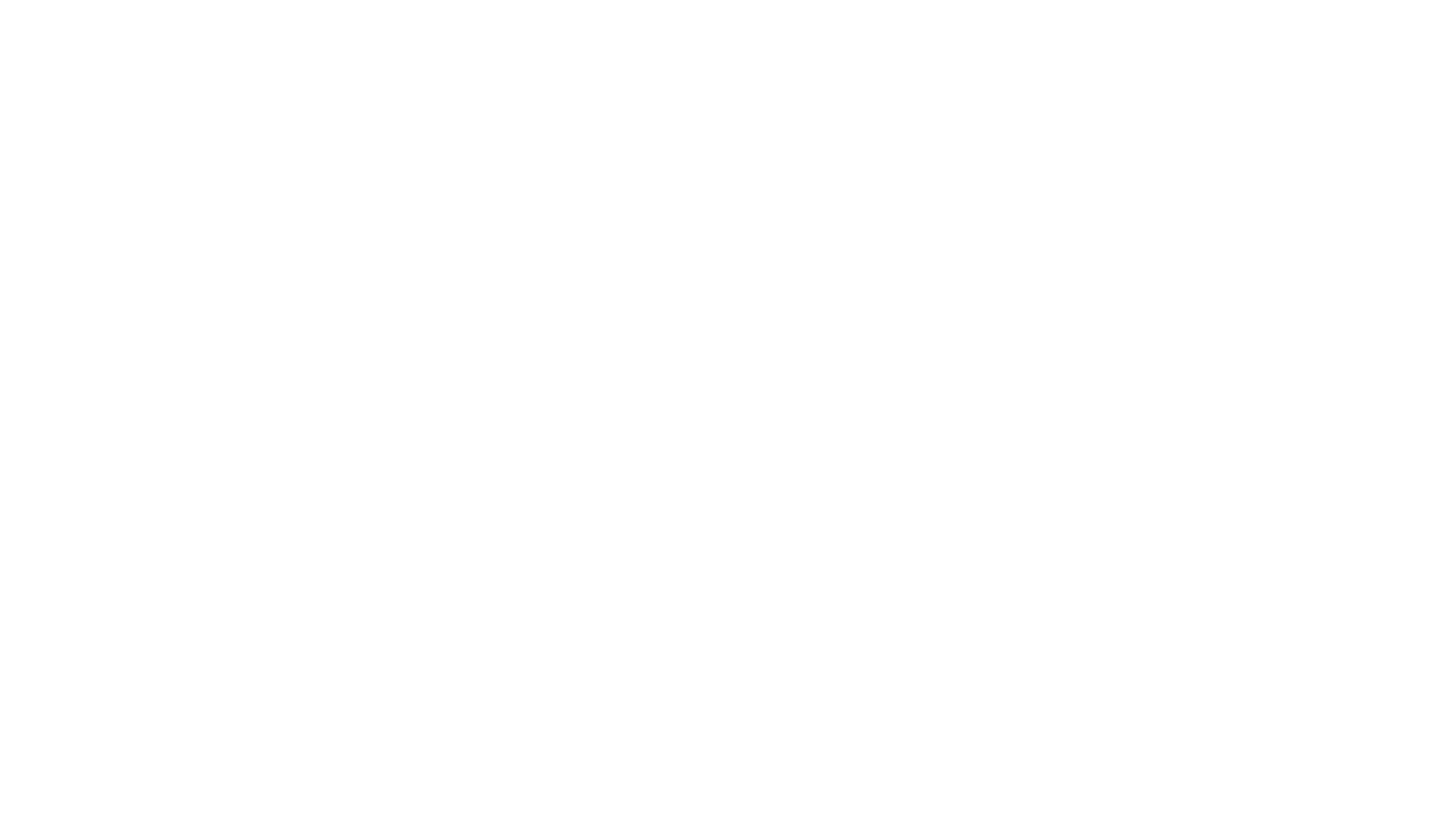

## Slide 3
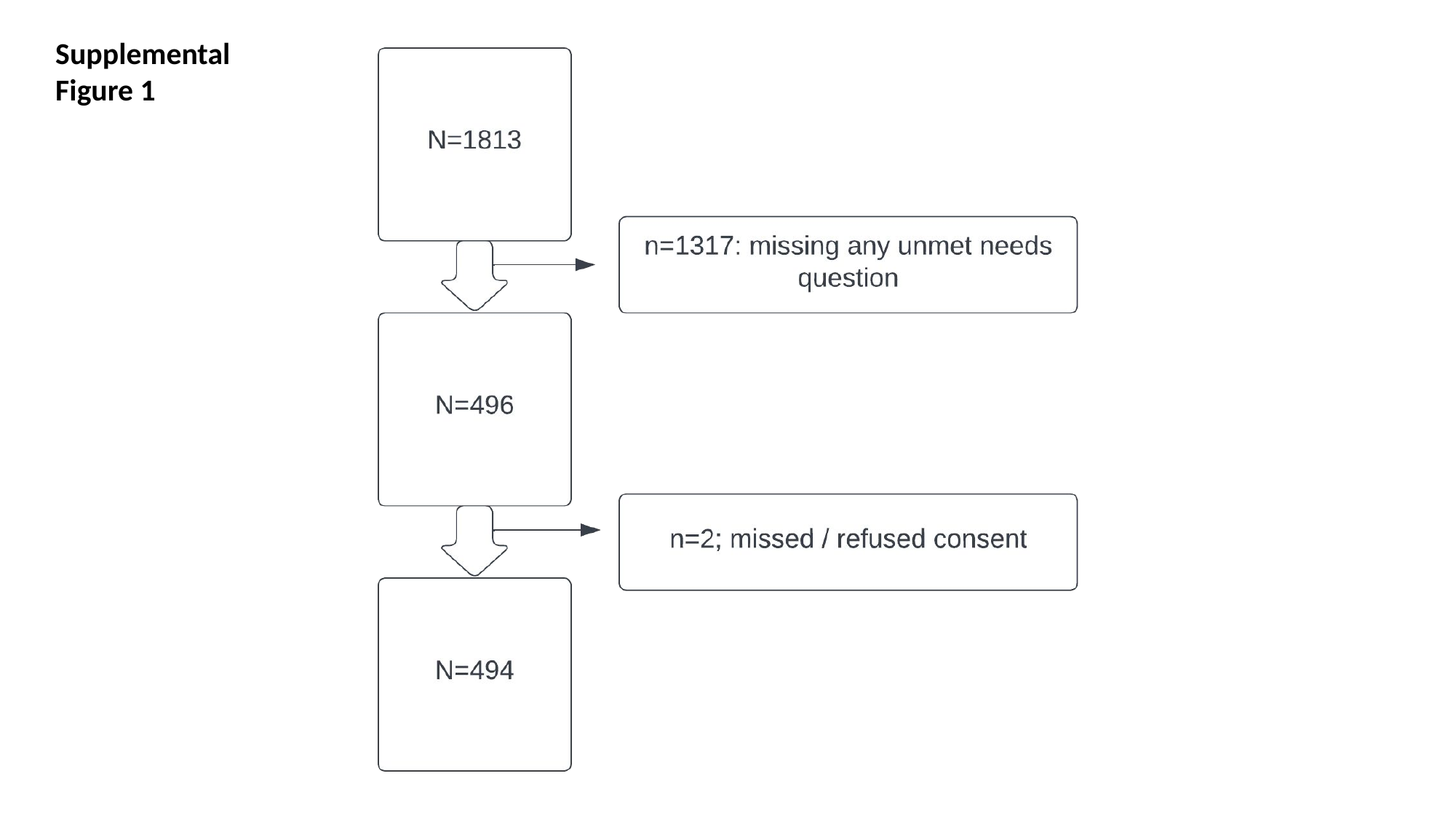

Supplemental
Figure 1

Supplement: Supplementary file 1 — Figure S1. [file CAM4-12-13846-s002.pptx]
