# Supplementary material for: Association of unmet basic resource needs with frailty and quality of life among older adults with cancer—Results from the CARE registry
Source: Cancer Med. 2023 May 28;12(12):13846–55. doi: 10.1002/cam4.6038 (PMC10315805; doi:10.1002/cam4.6038)
Supplement: Supplementary file 2 — Table S1. Table S2. Table S3. [file CAM4-12-13846-s001.docx]

**Association of Unmet Basic Resource Needs with Frailty and Quality of Life among Older Adults with Cancer – Results from the CARE Registry**

**SUPPLEMENTARY FILE**

**Contents:**

1. **Supplemental Table 1 ……………………………………………………………………. 2-3**
2. **Supplemental Table 2…………………………………………………………………….. 4-5**
3. **Supplemental Table 3…………………………………………………………………….. 6-8**

**Supplementary Table 2**: CARE Frailty Index items and prevalence of individual item impairment

| **Variable** | **n (%)** |
| --- | --- |
| Falls ≥1, 1 point | 82 (17.7) |
| Walk one block = limited a lot , 1 point | 228 (47.3) |
| IADL mobility (unable to/ with some help) , 1 point | 134 (27.6) |
| IADL shopping (unable to/ with some help) , 1 point | 139 (28.7) |
| IADL meal prepare (unable to/ with some help) , 1 point | 130 (26.8) |
| IADL housework (unable to/ with some help) , 1 point | 211 (43.4) |
| IADL medication (unable to/ with some help) , 1 point | 72 (14.9) |
| IADL money (unable to/ with some help) , 1 point | 60 (12.4) |
| ADL get in and out of bed (unable to/ with some help) , 1 point | 36 (7.4) |
| ADL dress (unable to/ with some help) , 1 point | 42 (8.6) |
| ADL bath (unable to/ with some help) , 1 point | 55 (11.4) |
| Global health, good =0.5 point | 179 (36.5) |
| fair/poor = 1 point | 204 (41.6) |
| Global quality of life, good =0.5 point | 189 (38.4) |
| fair/poor = 1 point | 132 (26.8) |
| Global physical health, good =0.5 point, | 175 (36.1) |
| fair/poor = 1 point | 203 (41.9) |
| Global mental health, good =0.5 point, | 183 (37.4) |
| fair/poor = 1 point | 82 (16.8) |
| Global satisfaction with social activities and relationship, good =0.5 point | 187 (38.1) |
| fair/poor = 1 point | 105 (21.4) |
| Global everyday activities, moderately=0.5 point | 191 (38.8) |
| a little/not at all = 1 point | 115 (23.4) |
| Global anxious/depression, sometimes =0.5 point | 100 (20.3) |
| often/always = 1 point | 111 (22.6) |
| Global fatigue, moderate =0.5 point, | 146 (29.7) |
| severe/very severe = 1 point | 78 (15.9) |
| Global pain, pain level 4-6 =0.5 point, | 192 (39.2) |
| pain level 7-10 = 1 point | 80 (16.3) |
| Global social activities and roles, good =0.5 point | 100 (21.2) |
| fair/poor = 1 point | 95 (20.1) |
| Weight loss 3 months or 6 months’ weight loss >=5%, 1 point | 188 (41.5) |
| Food intake less than usual, 1 point | 217 (45.5) |
| Activities and function (self-rated activity) ≥2 (in bed or chair less than half the day/ able to do little activity / Pretty much bedridden), 1 point | 130 (26.9) |
| Anxiety PROMIS T score >60, 1 point | 89 (18.6) |
| Depression PROMIS T score >60, 1 point | 60 (12.7) |
| Impaired Cognition, PROMIS T score <40, 1 point | 29 (5.9) |
| Number of daily medications ≥9, 1 point | 99 (20.8) |
| Social activity interference, Some of the time=0.5 point | 108 (22.2) |
| Most/ All of the time=1 point | 273 (56.2) |
| Eyesight Fair/Poor/totally blind, 1 point | 125 (25.9) |
| Hearing fair/Poor/Totally Deaf, 1 point | 156 (32.2) |
| Other Cancers or leukemia, 1 point | 104 (23.4) |
| Arthritis or rheumatism, 1 point | 163 (35.5) |
| Glaucoma, 1 point | 33 (7.3) |
| Emphysema or chronic bronchitis, 1 point | 47 (10.4) |
| High blood pressure, 1 point | 277 (60.0) |
| Heart disease, 1 point | 102 (22.9) |
| Circulation trouble in arms or legs, 1 point | 102 (22.1) |
| Diabetes, 1 point | 117 (25.7) |
| Stomach or intestinal disorders, 1 point | 144 (31.7) |
| Osteoporosis, 1 point | 45 (10.1) |
| Chronic liver or kidney disease, 1 point | 94 (20.5) |
| Stroke, 1 point | 37 (8.1) |

| **Supplemental Table 2. Multivariable logistic regression of the association between basic unmet needs and frailty.** | | |
| --- | --- | --- |
| **Variable** | **Unadjusted OR (95% CI)** | **Adjusted (95% CI)** |
| **Unmet need, yes vs. no** | **3.81 (2.36, 6.16)** | **3.27 (1.83, 5.86)** |
| **Age Group** |  |  |
| 60-64 |  | Ref |
| 65-69 |  | 0.86 (0.42, 1.75) |
| 70-74 |  | 1.24 (0.58, 2.67) |
| 75-79 |  | **2.54 (1.09, 5.93)** |
| 80+ |  | **3.04 (1.28, 7.25)** |
| **Sex, male vs. female** |  | 0.84 (0.51, 1.38) |
| **Race/Ethnicity** |  |  |
| Non-Hispanic White |  | Ref |
| Non-Hispanic Black |  | 1.75 (0.97, 3.17) |
| Other |  | 1.42 (0.47, 4.34) |
| **Education** |  |  |
| <High School |  | **3.40 (1.33, 8.68)** |
| High School |  | 1.75 (0.80, 3.82) |
| Some College |  | 1.54 (0.67, 3.57) |
| Associate's / Bachelor's Degree |  | 1.21 (0.51, 2.83) |
| Advanced Degree |  | Ref |
| **Employment Status** |  |  |
| Retired |  | Ref |
| Disabled |  | 2.05 (0.97, 4.29) |
| Part-time (<32hours/week) |  | 0.57 (0.13, 2.46) |
| Full-time (≥32hours/week) |  | 0.41 (0.16, 1.10) |
| Other |  | 0.55 (0.21, 1.10) |
| **Marital Status** |  |  |
| Single |  | 0.73 (0.29, 1.85) |
| Widowed/Divorced/Separated |  | 0.86 (0.50, 1.47) |
| Married |  | Ref |
| **Urban-Rural Status** |  |  |
| Urban |  | Ref |
| Rural |  | 0.42 (0.17, 1.04) |
| **Cancer Type** |  |  |
| Colorectal |  | Ref |
| Pancreatic |  | **3.54 (1.77, 7.08)** |
| Hepatobiliary |  | **3.56 (1.63, 7.77)** |
| Other |  | **2.09 (1.15, 3.79)** |
| **Cancer Stage** |  |  |
| 0-II |  | Ref |
| III |  | 0.77 (0.41, 1.45) |
| IV |  | 0.80 (0.45, 1.42) |

| **Supplemental Table 3. Multivariable logistic regression of the association between basic unmet needs and health-related quality of life (HRQoL).** | | |
| --- | --- | --- |
| **Physical HRQoL** | | |
| **Variable** | **Unadjusted OR (95% CI)** | **Adjusted (95% CI)** |
| **Unmet need, yes vs. no** | **2.90 (1.79, 4.71)** | **2.12 (1.19, 3.77)** |
| **Age Group** |  |  |
| 60-64 |  | Ref |
| 65-69 |  | 1.00 (0.52, 1.94) |
| 70-74 |  | 1.26 (0.63, 2.55) |
| 75-79 |  | 1.98 (0.86, 4.57) |
| 80+ |  | 1.09 (0.47, 2.56) |
| **Sex, male vs. female** |  | 0.64 (0.40, 1.02) |
| **Race/Ethnicity** |  |  |
| Non-Hispanic White |  | Ref |
| Non-Hispanic Black |  | 1.58 (0.88, 2.83) |
| Other |  | 1.64 (0.59, 4.60) |
| **Education** |  |  |
| <High School |  | **5.17 (2.03, 13.18)** |
| High School |  | 1.73 (0.79, 3.79) |
| Some College |  | **2.30 (1.02, 5.20)** |
| Associate's / Bachelor's Degree |  | 1.64 (0.71, 3.76) |
| Advanced Degree |  | Ref |
| **Employment Status** |  |  |
| Retired |  | Ref |
| Disabled |  | **2.58 (1.25, 5.31)** |
| Part-time (<32hours/week) |  | 1.23 (0.33, 4.60) |
| Full-time (≥32hours/week) |  | 0.48 (0.20, 1.11) |
| Other |  | 0.75 (0.32, 1.76) |
| **Marital Status** |  |  |
| Single |  | 0.67 (0.27, 1.67) |
| Widowed/Divorced/Separated |  | 0.91 (0.55, 1.53) |
| Married |  | Ref |
| **Urban-Rural Status** |  |  |
| Urban |  | Ref |
| Rural |  | 0.57 (0.26, 1.28) |
| **Cancer Type** |  |  |
| Colorectal |  | Ref |
| Pancreatic |  | **2.15 (1.12, 4.14)** |
| Hepatobiliary |  | **3.25 (1.54, 6.87)** |
| Other |  | 1.56 (0.91, 2.70) |
| **Cancer Stage** |  |  |
| 0-II |  | Ref |
| III |  | 1.29 (0.69, 2.40) |
| IV |  | 1.61 (0.91, 2.87) |
| **Mental HRQoL** | | |
| **Variable** | **Unadjusted OR (95% CI)** | **Adjusted (95% CI)** |
| **Unmet need, yes vs. no** | **3.09 (1.91, 5.01)** | **2.50 (1.43, 4.36)** |
| **Age Group** |  |  |
| 60-64 |  | Ref |
| 65-69 |  | 1.03 (0.55, 1.92) |
| 70-74 |  | 0.92 (0.47, 1.81) |
| 75-79 |  | 1.19 (0.53, 2.67) |
| 80+ |  | 0.88 (0.39, 2.01) |
| **Sex, male vs. female** |  | **0.56 (0.36, 0.89)** |
| **Race/Ethnicity** |  |  |
| Non-Hispanic White |  | Ref |
| Non-Hispanic Black |  | 1.63 (0.93, 2.85) |
| Other |  | 0.75 (0.26, 2.18) |
| **Education** |  |  |
| <High School |  | **2.74 (1.15, 6.51)** |
| High School |  | 1.70 (0.84, 3.45) |
| Some College |  | 1.58 (0.74, 3.35) |
| Associate's / Bachelor's Degree |  | 1.04 (0.48, 2.22) |
| Advanced Degree |  | Ref |
| **Employment Status** |  |  |
| Retired |  | Ref |
| Disabled |  | 1.49 (0.75, 2.95) |
| Part-time (<32hours/week) |  | 0.54 (0.14, 2.04) |
| Full-time (≥32hours/week) |  | **0.28 (0.12, 0.66)** |
| Other |  | 0.80 (0.37, 1.76) |
| **Marital Status** |  |  |
| Single |  | 0.71 (0.31, 1.63) |
| Widowed/Divorced/Separated |  | 0.76 (0.46, 1.25) |
| Married |  | Ref |
| **Urban-Rural Status** |  |  |
| Urban |  | Ref |
| Rural |  | 0.55 (0.25, 1.21) |
| **Cancer Type** |  |  |
| Colorectal |  | Ref |
| Pancreatic |  | 1.68 (0.89, 3.17) |
| Hepatobiliary |  | **2.79 (1.35, 5.78)** |
| Other |  | 1.48 (0.88, 2.48) |
| **Cancer Stage** |  |  |
| 0-II |  | Ref |
| III |  | 1.00 (0.55, 1.81) |
| IV |  | 1.29 (0.75, 2.20) |
